# Permeability of Ciprofloxacin-Loaded Polymeric Micelles Including Ginsenoside as P-glycoprotein Inhibitor through a Caco-2 Cells Monolayer as an Intestinal Absorption Model

**DOI:** 10.3390/molecules23081904

**Published:** 2018-07-31

**Authors:** Behzad Sharif Makhmal Zadeh, Golbarg Esfahani, Anayatollah Salimi

**Affiliations:** Nanotechnology Research Center, School of Pharmacy, Ahvaz Jundishapur University of Medical Sciences, Golestan Ave, Ahvaz 67123, Iran; golbarg_isfahani@yahoo.com (G.E.); anayatsalimi2003@yahoo.com (A.S.)

**Keywords:** ciprofloxacin, ginsenoside, P-glycoprotein, gastrointestinal drug transporter, polymeric micelles

## Abstract

The low oral bioavailability of ciprofloxacin is associated with two distinct challenges: its low aqueous solubility and efflux by p-glycoproteins (P-gp) in the intestinal membrane. Several studies were conducted in order to improve its solubility and permeability through the gastrointestinal membrane. In this study, in a full factorial design study, eight polymeric micelles were prepared and their characteristics, including particle size, loading and release rate were evaluated. Polymeric micelles demonstrated particle sizes below 190 nm and 27–88% loading efficiency. Drug release was affected by drug solubility, polymeric micelle erosion and swelling in simulated gastrointestinal fluids. An optimized polymeric micelle was prepared based on appropriate characteristics such as high drug loading and low particle size; and was used for a permeation study on Caco-2 cells. Optimized polymeric micelles with and without ginsenoside and ginsenoside alone enhanced drug permeability through Caco-2 cells significantly in the absorptive direction. The effect of ginsenoside was dose dependent and the maximum effect was seen in 0.23 mg/mL concentration. Results showed that P-gp may not be responsible for ciprofloxacin secretion into the gut. The main mechanism of ciprofloxacin transport through Caco-2 cells in both directions is active diffusion and P-gp has inhibitory effects on ciprofloxacin permeability in the absorptive direction that was blocked by ginsenoside and micelles without ginsenoside.

## 1. Introduction

Ciprofloxacin is a wide spectrum antibiotic approved by the U.S. Food and Drug Administration (FDA) for 14 infections, especially urinary and respiratory infections such as acute uncomplicated bladder, chronic bacterial prostate and upper respiratory tract infections [1]. The oral bioavailability of ciprofloxacin is about 59 ± 60% [2,3] and it is associated with two distinct challenges: (1) solubility in gastrointestinal fluids; (2) efflux by p-glycoprotein (P-gp) in the intestinal membrane [4,5]. Ciprofloxacin is a zwitterion, which means its aqueous solubility is affected by pH. The maximum aqueous solubility of ciprofloxacin occurs at pH <5 or pH >10. Therefore precipitation of this drug in the small intestine may occur [1,6]. Transport data in P-gp overexpressing cell lines such as MDCK1 and MDCK1-MDR1 that is originated from transfection of Madin Darby Canine kidney cells with the MDR1 gene [7] and Caco-2 [8] indicate that ciprofloxacin is a substrate of P-gp. P-glycoprotein, also known as multidrug resistance protein (MDR), is an important cell membrane protein that pumps many foreign substances out of cells [7,8]. The decrease in oral bioavailability of ciprofloxacin due to the p-glycoprotein efflux in the intestine has been demonstrated by several studies [9,10]. P-glycoprotein is an efflux transporter located in the apical membrane acting as a biological barrier by eliminating toxins and xenobiotics such as drugs out of cells [3]. Inhibition of P-gp is an effective strategy for oral drug bioavailability improvement. The effect of P-gp inhibitors in improvement of oral drug absorption has been demonstrated by several studies on different drugs [11,12,13]. The inhibition of P-gp is mainly done by three mechanisms: (i) competitively or non-competitively blocking the drug binding site; (ii) changing the integrity of the cell membrane lipids; (iii) intervention on ATP hydrolysis [14,15,16]. Three generations of P-gp inhibitors are introduced. The first group includes pharmacologically active compounds such as verapamil, cyclosporine and *trans*-flupenthixol [17]. Utilization of these substances is associated with potential toxicity at the doses that are needed to inhibit P-gp [18]. The second group, which inhibit the cytochrome PA4 enzyme, demonstrates lower pharmacological activities and higher P-gp affinity. The components in the third group such as tariquidar inhibit P-gp with higher affinity and lower toxicity [19]. Ginsenosides are the main active components of ginseng that indicate a P-gp inhibitory effect [20]. He et al. suggested that ginsenoside RC, Rb1, Rb2 and quercetin inhibited P-gp-mediated digoxin transport at 100 μM in a Caco-2 cell model [21]. 20(*S*)-Ginsenoside Rg3 (G-Rg3) is a red ginseng saponin that at 20–120 μM concentration decreases the membrane fluidity, thereby blocking drug efflux [22]. On the other hand, Kim et al. reported that G-Rg3 competed with drugs for binding to P-gp thereby blocking drug efflux [23]. Designs and formulations which can inhibit the P-gp efflux pump and improves ciprofloxacin solubility simultaneously may be useful in increasing oral bioavailability. A few approaches have been used for increasing ciprofloxacin oral bioavailability. Breda et al. prepared a novel high soluble aluminium:ciprofloxacin complex that decreased the dose:solubility ratio of ciprofloxacin at pH 6.8–7 below 100 mL [24].

Polymeric micelles are self-assembled core–shell nanostructures formed in an aqueous solution consisting of amphiphilic block copolymers such as poloxamers that can entrap hydrophilic and hydrophobic compounds and charged macromolecules through electrostatic, hydrophobic, and hydrogen bonding interactions [25] and the stereo complex formation [26] Polymeric micelles as functional polymer systems are able to modify the physical characteristics of drug such as solubility by their nanometer particle size, if they are simply prepared and non-irritant [27,28]. Polymeric micelles enhance drug absorption by four mechanisms: (1) controlled release of the drug at the target site (2) protection of the drug from destruction in the gastrointestinal tract (3) increased residence time in the gut and (4) inhibition of efflux pumps [29,30]. Polymeric micelles demonstrate thermodynamic and kinetic stability upon dilution and preserve the stable core-shell structure in the gastrointestinal tract [31]. Amphiphilic polymers inhibit of efflux transport with a modification of the fluidity of the cellular membrane [32]. Pluronic block copolymers (poloxamers) including ethylene oxide and propylene oxide induce alteration in microviscosity of cell membranes that is attributed to the change in the lipid bilayer structure [33].

In this study, we have designed poloxamer/phosphatidylcholine/cholesterol polymeric micelles, which contain G-Rg 3 as a P-glycoprotein inhibitor and investigated some properties of these drug carriers, including their efficiency in solubilizing and retaining the ciprofloxacin, and their ability to bypass the P-glycoprotein mediated efflux in the intestine using a Caco-2 cell monolayer as an intestinal absorption model. In this study, lecithin and cholesterol were used as the lipid core of the polymeric micelles. Previously, Varshosaz et al. prepared polymeric micelles with a lipid core of cholesterol for docetaxel delivery to B16F10 melanoma cells [34]. The presence of cholesterol as a lipid core decreased the CMC of the polymeric micelles. In another study a docetaxel-loaded lecithin-stabilized micellar drug delivery system for improving the therapeutic efficacy [35]. In these and similar studies, lecithin and cholesterol have been used as lipid cores for increasing the hydrodynamic diameter and thus drug loading and for increasing drug permeability through biological membranes. Labrafil (oleoyl polyoxyl-6 glycerides) and labrasol (caprylocaproyl polyoxyl-8 glycerides) are used as non-ionic surfactant for the formation of micelles.

## 2. Results and Discussion

### 2.1. Critical Micelle Concentration (CMC) Results

In this experiment CMC determines is a criterion of micelle formation. For this purpose plots of surface tension against concentration with and without polymer are drawn (Supplementary Materials). The break in surface tension against concentration is considered as the CMC. The CMC values for a mixture of surfactants with and without polymer were 0.06 ± 0.003 and 0.073 ± 0.004 mg/mL, respectively. The results indicate polymer addition to the labrasol and labrafil mixture decreased the CMC value significantly (*p* ˂ 0.05), which increases the micelle stability, since the critical micelle concentration is achieved at a lower concentration of surfactant (less than 135 mg/mL). Polymer increased the micelle formation tendency of labrafil and labrasol blend. Therefore the formed polymeric micelle would be more resistant to dilution effects in the GI tract and the micelle would be more stable [36]. The effect of ciprofloxacin on CMC was also evaluated in this research. For this purpose, the CMC was calculated with and without ciprofloxacin, which showed no significant differences induced by ciprofloxacin.

### 2.2. Formulation Characterization

#### 2.2.1. Particle Size and Encapsulation Efficiency (EE%)

Different polymeric micelles components based on factorial design and formulation characteristics including particle size and EE% are presented in Table 1. Particle size and EE% are the main characteristics that influence on the drug delivery properties of polymeric micelles. The regression equations for the model relating particle size (PZ) and EE% are shown by Equations (1) and (2), respectively. The different sizes of particles were between 109 to 183 nm with a polydispersity index of less than 0.5. A study of the influence of the independent variables on mean particle size indicated significant and antagonistic effects of ginsenoside concentration (*p* = 0.001), CMC (*p* = 0.009) and the interaction CMC×drug concentration (D) (*p* = 0.03) on particle size.

EE% was in the range of 27.9 to 87.9%. Maximum and minimum amounts of EE% were presented by formulations number 4 and 7, respectively. Different carriers including lipid-based, chitosan-based, and albumin-based carriers were used for ciprofloxacin encapsulation. They provided an EE% of 30.7%, 35%, and 40%, respectively. Gelatin nanocarriers were also designed and evaluated but they did not show suitable loading [37], therefore we conclude that polymeric micelles show better drug loading than other carriers.

**Figure 1:**
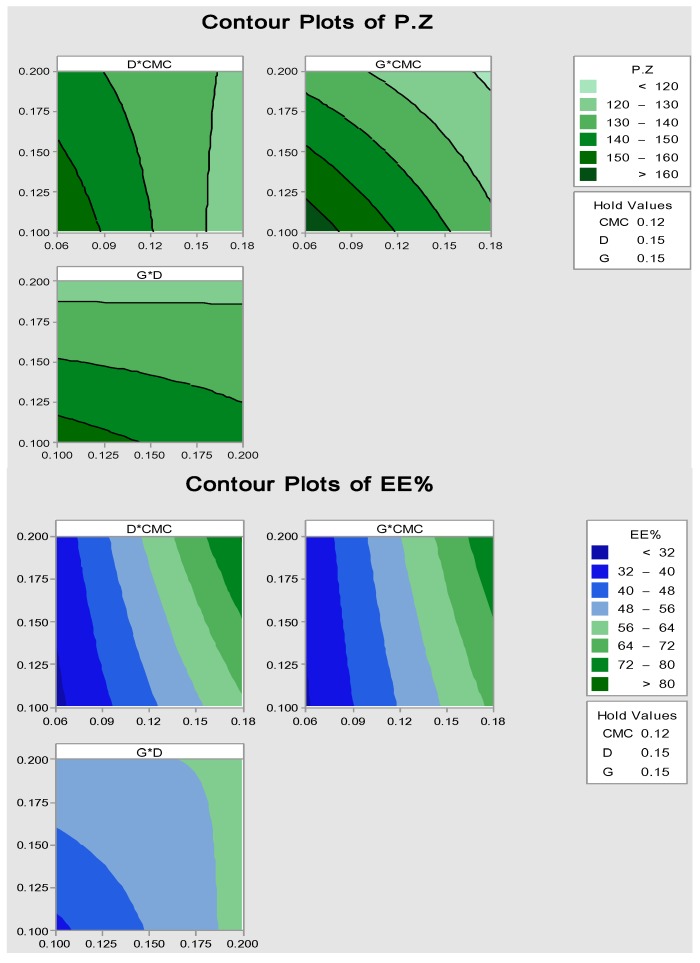
Contour plots of particle size (P.Z) and EE% (the *X* and *Y* axes present different amount of independent variable such as concentration of ciprofloxacin (D), concentration of ginsenoside R-g3 (G) and concentration of surfactant (CMC)).

Regression analysis indicated significant and synergistic effects of CMC (*p* = 0.022), D (*p* = 0.03) and G (*p* = 0.001) on EE%. The results showed an increase in CMC, D and G, caused a significant increase in EE%. Based on these results, G-Rg3 may increase the drug solubility in polymeric micelles and, thus, increase drug loading. It seems that polymeric micelles have higher capacity for drug loading when a higher drug concentration is used. It was reported that polymeric micelles increased ciprofloxacin solubility in aqueous environment with different pH values [38], therefore, it seems that polymeric micelles increase ciprofloxacin aqueous solubility by providing a sufficient amount of EE%. Contour plots of the relation between the independent variables and P.Z, EE% are shown in Figure 1.

P.Z = 340.3 − 1121 (CMC) − 938 (G) + 4600 (CMC × D)(1)

EE% = 126 − 1189(CMC) − 724(D) − 734(G) + 9247(CMC × D) + 8987(CMC × G) + 4676(D × G) − 54,096(CMC × D × G)(2)

#### 2.2.2. Release Study in Simulated Gastric Fluid (SGF) and Simulated Intestine Fluid (SIF)

Percentage of released drug is an important characteristic of the formulation which plays an important role in formulation effectiveness. Drug release profiles for each formulation were provided in SGF and SIF.

**Figure 2:**
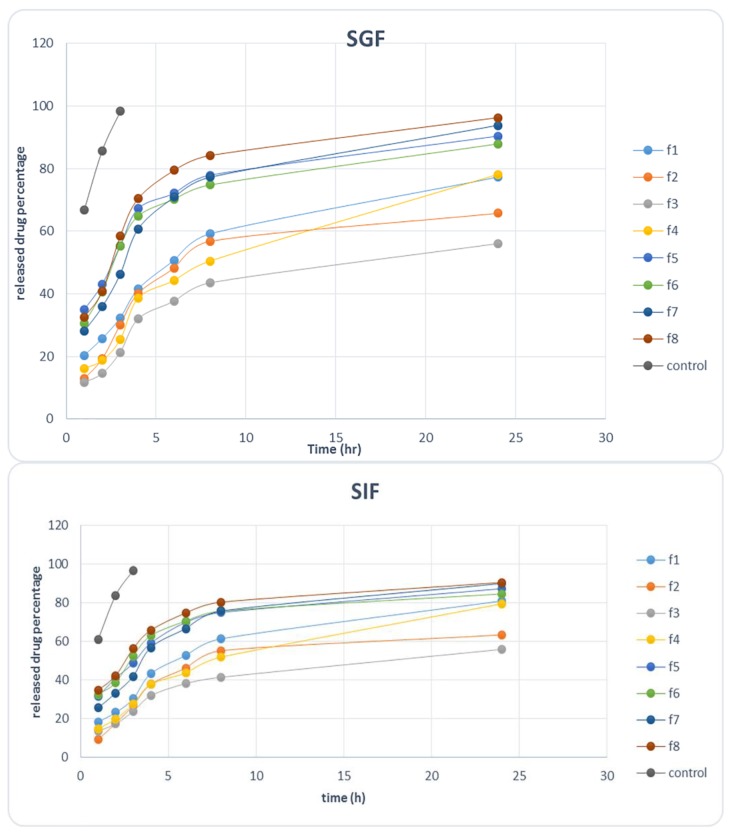
Released ciprofloxacin percent against time in SGF and SIF solutions.

The percentage of ciprofloxacin-released profiles in SGF and SIF solutions are presented in Figure 2. Percent of drug released after 4 h (D4%) was used as a sign of drug amount in the main site of absorption (small intestine) because 50% of the stomach contents are emptied after 2.5 to 3 h [39]. In SGF D4 % was between 32.1% and 70%, and in SIF it was between 29.3–67.68%.

No significant difference was found between D4% values in both solutions Here, in both solutions regression analysis indicated the significant and antagonistic effects of surfactant + cosurfactant concentration (*p* = 0.001–0.0012) and also G-Rg3 concentration (*p* = 0.024–0.003) on D4%. Therefore, an increase in surfactant+cosurfactant concentration and G-Rg3 concentration resulted in a significant decrease in D4%. It seems that increase in these two independent variables enhances the ciprofloxacin solubility and loading in micellar formulations. In a release study, released drug percentage after 24 h (D24%) was calculated in order to attain the release mechanism, but as the drug would pass the whole GI tract during 24 h, D24% results are useless. Comparing the released drug results with control demonstrates that almost all drug amounts in control are released and penetrate the acetate cellulose membrane. This indicates that the acetate cellulose membrane had not been the limiting step and the limiting step was the drug release from polymeric micelle formulations in fact. Therefore, the calculated rate was the drug release rate not the drug penetration rate through acetate cellulose membrane.

For both solutions drug released data were fitted to different kinetic models and it was found that the Weibull model is the best model in describing ciprofloxacin release rate from polymeric micelles. Correlation coefficient and regression *p*-value are criteria sued for the evaluation of the power of a kinetic model in precisely assessing the relation between the released drug percentage and time.

A higher correlation coefficient value indicates a higher suitability of the kinetic model. Based on the obtained results, the Weibull model showed the maximum correlation coefficient (R^2^) value (0.983) among all models. This is a practical model used for describing the fast and long drug release from carrier. This mechanism of release indicates that the drug release is affected by different mechanisms and Fickian and non-Fickian behaviors are important in the drug release [40]. On the other hand, it is stated that the Weibull model describes release in a complicated way in which swelling and polymer erosion play an important role [41]. Therefore, ciprofloxacin release of the polymeric micelle formulation follows a complicated mechanism in which swelling, polymer erosion and solubility all may play an important role. In this section, G-Rg3 released percent was calculated. Based on release data (not shown) 69–94% of G-Rg3 was released after 8 h.

### 2.3. Optimized Formulation

#### 2.3.1. Characterization of Optimized Polymeric Micelle

**Figure 3:**
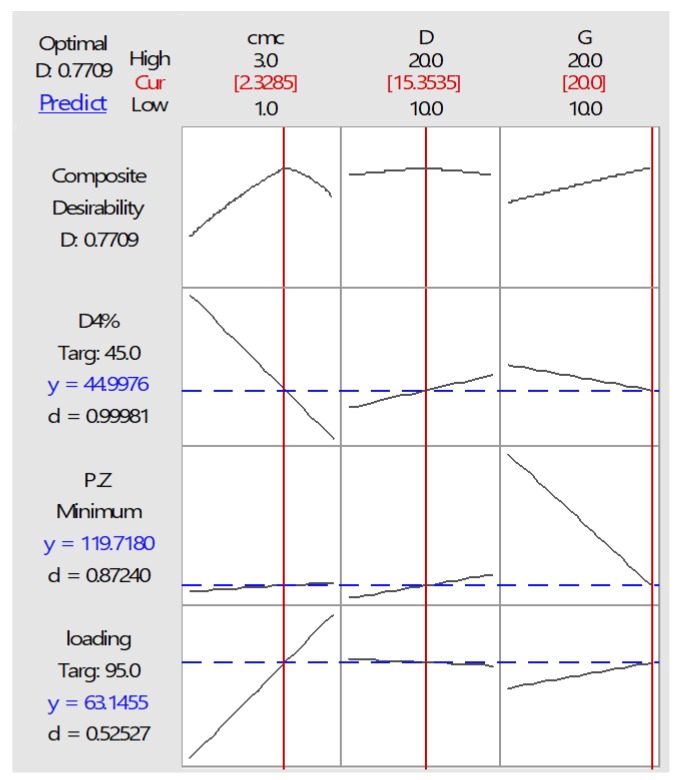
Desirability plot of optimize polymeric micelle formulation (representative optimal amounts of surfactant, ciprofloxacin and G-Rg3 for providing desired values of D4%, particle size (P.Z) and drug loading (EE%).

Based on contour plots, and after ANOVA analysis, numerical optimization was applied for getting the final optimized polymeric micelle with desired responses like minimum particle size, maximum EE% and 40–50% D4%. Composite desirability of the optimized bath was calculated to be 0.773 and it is shown in Figure 3.

The optimized batch components and its actual and predicted particle size and EE% are tabulated in Table 2. Therefore, optimized formulation was prepared with three independent optimized variables, including 2.33 CMC, 20 mg G-Rg3 (0.2 mg/mL) and 15.35 mg ciprofloxacin (0.153 mg/mL), which are shown in Table 3. Then, characteristics such as particle size, EE%, and D4% were measured and compared with the predicted values. The difference between actual and predicted values of dependent variables was evaluated by *t*-test and *p*-values are reported in Table 4. Predicted values were calculated based on equations that indicate the correlation between dependent and independent variables. Based on the results and at 5% significance, there was no significant difference between the actual and predicted value.

In addition, the thermal behavior of the optimized polymeric micelles and permeability parameters through Caco-2 cells were studied.

#### 2.3.2. Optimized Polymeric Micelle Stability

• Micelle stability in refrigerated temperature

Ciprofloxacin-loaded optimized polymeric micelles that were stored at 4 °C for 3 months indicated less than a 4% increase in particle size that was not significant. Therefore, it seems that the optimized batches have perfect stability. On the other hand, ciprofloxacin content after 3 months decreased less than 4.2% ± 0.5. This evidence indicates the ability of optimized formulation to protect ciprofloxacin against environmental stress.

• Micelle stability in media modeling physiological conditions

Results indicated that in SGF and SIF without bile salt, size of micelle and drug content didn’t change significantly, but micelles incubated with SGF/bile salt and SIF/bile salt mixtures demonstrated 10.28 (%) ± 1.1 and 7.60 (%) ± 0.65 decreases in micelle size, respectively. On the other hand, no any drug precipitation was found, which is a sign of the stability of ciprofloxacin-loaded polymeric micelles.

#### 2.3.3. Differential Scanning Calorimetry of Optimized Formulation

DSC experiments were performed to study the interaction between ciprofloxacin and polymeric micelle components and evaluate the ciprofloxacin physical state in the optimized polymeric micelles. Thermotropic states of ciprofloxacin powder, optimized ciprofloxacin-loaded polymer micelle, and placebo optimized polymer micelle were evaluated by comparing their mean transition temperatures (Tm). Their enthalpies (∆H) are tabulated in Table 3.

According to the DSC thermograms, the optimized formulation shows three different peaks at −20 °C, 0 °C and 140 °C. A peak in 260 °C was observed in the ciprofloxacin powder thermogram, which is related to the ciprofloxacin melting point, but this peak was not seen in the optimized formulation thermogram, therefore, the polymeric micelle formulation could solubilized all the drug amount.

On the other hand, the optimized formulation without G-Rg3 also showed three peaks at −20, 0 and 140 °C. The peak appearing at −20 °C is related to the freezing of interfacial water (water bonded to the surfactant film which is not free in the polymeric micelle structure) with an enthalpy of 189 mJ/mg. this peak is also seen in the thermogram of the blank formulation without G-Rg3 with a similar enthalpy of 220 mJ/mg, while in the optimized formulation thermogram, this peak shows a much higher enthalpy of 742 mJ/mg. Therefore, this significant difference in enthalpy is probably caused by the G-Rg3 effect on interfacial water freezing, whereas ciprofloxacin didn’t show this effect. It seems that water freezing enthalpy was increased by bonding between ginsenoside and water. This behavior is also seen in the bulk water melting point in 0 centigrade degrees. G-Rg3 decreased the needed energy for bulk water melting by bonding to bulk water and decreasing the amount of bulk water, whereas this effect wasn’t seen with the ciprofloxacin [42].

#### 2.3.4. Optimized Formulation Permeability through Caco-2 Cells

Permeability parameters, including permeated drug after 4 h (%Q_4_) and permeability coefficient (P) for AP-BL and BL-AP directions are tabulated in Table 4. AP-BL indicates drug absorption from intestine to blood and BL-AP demonstrates drug secretion from blood into the intestine mostly by P-gp. BL-AP is a sign of a barrier against drug absorption. Three controls were prepared by dissolving 0.14 mg/mL ciprofloxacin (equal to the drug concentration in the optimized formulation) and different concentrations of G-Rg3 (lower and higher than concentration of ginsenoside in optimized formulation) in aqueous solution of 0.018 mg/mL of labrasol + labrafil (equal to 0.3 CMC).

Based on the obtained results, in the AP-BL direction it seems that permeability through Caco-2 cells is influenced by the G-Rg3 concentration and polymeric micelle formation. Maximum %Q4 and P were created by control 3 and the optimized formulation with no significant difference between them. Obtained *p* values in this study agreed with to previously reported values [43]. Removing G-Rg3 from the optimized formulation caused lower Q4% and P than in the optimized formulation and control 3, higher values than control 1 and equal values to control 2. Therefore, the optimized formulation was able to increase ciprofloxacin permeability on its own, and its effect is equal to 0.23 mg/mL G-Rg3 alone. Permeability enhancement effect of G-Rg3 with the concentration of 0.23 mg/mL is equal to optimized formulation and higher than G-Rg3 with the concentration of 0.08 mg/mL. Therefore, the permeability enhancement effect of ginsenoside is dose dependent. Comparison of permeability parameters between AP-BL with BL-AP directions showed that increasing the G-Rg3 concentration did not increase ciprofloxacin P and Q4% in the BL-AP direction. This means that P-gp may not be responsible for ciprofloxacin secretion into the gut. In the BL-AP direction maximum Q4% and P were provided by the optimized formulation with and without G-Rg3, which means other transporters that are influenced by polymeric micelles are important in ciprofloxacin secretion. A similar result was reported for methylprednisolone loaded in polymeric micelles formed by a polyethylene oxide chain polymer [44]. In the present study, micelles were formed by poloxamer, which is a nonionic triblock copolymer composed of polyoxypropylene-polyoxyethylene. The effect of poloxamer on P-glycopropein-mediated efflux by energy depletion and membrane fluidity in the blood-brain barrier has been reported previously [45], but the effect of poloxamer on other transporters has not been reported. Based on the present result it seems that poloxamer can inhibit ciprofloxacin transporter and thus increase BL-AP permeability, therefore the main mechanism of ciprofloxacin transport through Caco-2 cells in both directions is active diffusion and. Pg-Protein carriers have an inhibitory effect on ciprofloxacin permeability in the AP-BL direction that was blocked by G-Rg3. The P and Q4% values in the BL-AP direction were significantly more than values in the AP-BL direction, which is similar to a result that was demonstrated previously [43].

## 3. Materials and Methods

### 3.1. Materials

Ciprofloxacin (cipro) powder was purchased from Daru Pakhsh Company (Tehran, Iran). Oleic acid, propylene glycol (PG), cholesterol, poly ethylene glycol (PEG) and poloxamer F127 (poloxamer) were obtained from Merck (Berlin, Germany). Lecithin was purchased from Sigma-Aldrich company (Berlin, Germany). Caprylocaproyl polyoxyl-8 glycerides (Labrasol^®^) and oleoyl polyoxyl-6 glycerides NF (Labrafil^®^) were a gift from GATTEFOSSE Company (Paris, France). Cellulose acetate membrane was purchased from the Betagen Company (Tehran, Iran).

Caco-2 cells were purchased from the Iranian Biological Research Center (Tehran, Iran). Dulbecco’s modified eagle’s medium (DMEM) and fetal bovine serum (FBS) were purchased from Gibco Bioscience company (Dublin, Ireland). Penicillin-streptomycin, six well Transwell culture plates (pore size 0.45 μm, diameter 24 mm) were obtained from Sigma-Aldrich. G-Rg3 was obtained from Gol Daru Herbal Company (Esfahan, Iran) as a gift. Simulated gastric fluid (SGF) and simulated intestinal fluid (SIF) were prepared based on the United States Pharmacopeia (USP 29) procedure. SGF was made by dissolving 2 g of sodium chloride and 3.2 g of purified pepsin (purchased from Sigma-Aldrich) in 7 mL of hydrochloric acid and sufficient water to make 1000 mL and adjust pH = 1.2. SIF was also prepared by dissolving 6.8 g of monobasic potassium phosphate in 250 mL of water mixed and 77 mL of 0.2 N sodium hydroxide and 500 mL of water were added. Then, 10 g of pancreatin (purchased from Sigma-Aldrich) was added and mixed and the pH was adjusted at 6.8 and diluted with water to 1000 mL. All chemicals and solvents were of analytical grade. Fresh double distilled water was used in the experiments. Minitab15 software was used for experimental design and the evaluation of the effects of variables on responses.

### 3.2. Methods

#### 3.2.1. Ciprofloxacin Analysis by HPLC

HPLC was carried out on a Waters C18 (25 cm, 4.6 mm) column (Waters, Milford, CT, USA) with a mixture of acetate buffer (pH = 2.8) and acetonitrile (70:30) as mobile phase at a flow rate of 1.5 mL/min. Detection was carried out at 278 nm. Injection volume was 100 μL. All samples were analyzed in triplicate.

#### 3.2.2. Determination of Critical Micelle Concentration (CMC)

CMC calculation was done by surface tension measurement method. For this purpose surfactant and co-surfactant (labrasol + labrafil) aqueous solutions with different concentration and constant concentration of polymer were prepared. Surface tension of these solutions was measured at 25 °C with a Torsion balance (WHITE ELEC Model NO. 83944E, Torsion Balance Supplies, North Somerset, England). Then, chart of surface tension versus log concentration was plotted. There is a break in surface tension at a concentration associated with critical micelle concentration (CMC), which corresponds to the onset of micelle formation on the bulk solution.

#### 3.2.3. Optimization by Factorial Statistical Design

In the present study, factorial design was used for optimization based on preliminary experimental observation. Surfactant +co-surfactant concentration based on CMC, ciprofloxacin and G-Rg3 concentrations were used as three independent variables in two levels. Ciprofloxacin concentrations were selected based on the recommended dose of ciprofloxacin reported by WHO. The effects of independent variables on different micelle characteristics were evaluated.

#### 3.2.4. Micelle Formation and Drug Loading

Appropriate amounts of ciprofloxacin, lecithin, cholesterol, poloxamer, ginsenoside R-g3, oleic acid and half percent of labrasol-labrafil (1:1) were accurately weighed and dissolved in 10 mL chloroform as a solvent in a round-bottom flask. Then the solvent was removed by vacuum distillation with a rotary evaporator (Heidolph, Schwabach, Germany) at 65 °C and 120 rpm. The residual amount of solvent was removed under vacuum overnight room temperature. The flask was then warmed at 45 °C (natural lipid glass transition temperature) [46,47] and the aqueous phase including PG, PEG, half percent of labrasol-labrafil (1:1), and water was added to and stirred for 30 min. Then the resulting solution was shaken for 3 min and diluted to 20 mL.

#### 3.2.5. Characterization of Polymeric Micelles

##### Determination of Average Particle Size

Particle size was measured at 25 °C by particle size analyzer. The mean droplet size of samples was determined at by SCATTER SCOPE 1 QUIDIX (Seoul, Korea) based on photon correlation spectroscopy with a wide measurable size range (1–7000 nm). Each sample was measured in triplicate.

##### Encapsulation Efficiency (EE%)

Ciprofloxacin EE% was determined by the ultrafiltration method to separate free ciprofloxacin from polymeric micelle solution. A polymeric micelle solution with a defined amount of ciprofloxacin was added into centrifugal-ultrafiltration tubes (Microcon MWCO 3000, Millipore Co., Vernon Hills, IL, USA) and centrifuged at 25,000 rpm for 30 min at 4 °C. Then, supernatant was analyzed by HPLC for unloaded ciprofloxacin. The amount of loaded ciprofloxacin was calculated by subtracting the unloaded ciprofloxacin from initial ciprofloxacin added to the polymeric micelle. EE% was then measured by Equation (3):

% encapsulation = (cipro total amount − cipro amount in supernatant)/cipro total amount × 100(3)

#### 3.2.6. Micelle Stability

• Micelle stability in refrigerator condition:

The drug-loaded micelles were stored at 4 °C for 3 months. The physical stability of polymeric micelles was evaluated by monitoring the time-dependent changes in the physical characteristics such as drug precipitation and change in micelle particle size.

• Stability in media modeling physiological conditions and effect of dilution

The stability of 1 mL ciprofloxacin-loaded polymeric micelles was studied by incubation in 1, 10 and 50 mL buffer phosphate, pH 7.4; SGF, pH 1.2 and SIF, pH 6.8 with and without bile salts (5 mM) for 12 h at room temperature [48]. Then at defined time intervals polymeric micellar solutions were filtered through a 0.2 μm membrane filter. The particle size, taken as a sign of physical stability and ciprofloxacin content as a sign of chemical stability were analyzed and compared with ciprofloxacin solution as a control.

#### 3.2.7. In Vitro Release Kinetics of Ciprofloxacin

Ciprofloxacin-loaded polymeric micelles were evaluated for in vitro release of ciprofloxacin by dialysis bag technique at 37 °C. Polymeric micelles including a defined amount of ciprofloxacin were placed in cellulose acetate dialysis membrane with a molecular weight cut off 3000–4000 Da, and dialyzed against simulated gastric fluid (SGF) and simulated intestinal fluid (SIF). The release medium was stirred at 50 rpm. At time intervals 1, 2, 4, 6, 8, 24 and 48 h 2 mL samples were withdrawn and replaced with an equal volume of fresh medium. The samples were analyzed for ciprofloxacin concentration by HPLC. Experiments were run in triplicate for each time point of release kinetics. Results are expressed as percentage of ciprofloxacin released through polymeric micelles over time. In this experiment, the same concentration of ciprofloxacin in SGF and SIF were used as a control.

#### 3.2.8. Differential Scanning Calorimetry (DSC) Analysis

The thermal behavior of optimized polymeric micelle was performed using a Mettler (Mettler Toledo Company, Mississauga, ON, Canada) differential scanning calorimetric (DSC) system. Samples at first heated to 50 °C and kept at this temperature for 5 min to remove their thermal history. Then, the temperature was reduced to 0 °C with 5 °C/ min. The samples were kept at 0 °C for 5 min, and the temperature was then increased to 160 °C with the same rate. The possible incompatibility between drug and carrier was also evaluated by measuring transition temperature and enthalpy.

#### 3.2.9. Cell Culture

The human colon adenocarcinoma cell line (Caco-2) was obtained from the Iranian Biological Research Center (Tehran, Iran). Caco-2 cells had a passage of 20–30 and were maintained in Dulbecco’s modified Eagle’s medium, containing 10% fetal bovine serum, 2 mM of l-glutamine, penicillin (100 U/mL) and streptomycin (100 U/mL) in an atmosphere of 5% CO_2_ and 90% relative humidity at 37 °C.

#### 3.2.10. Permeation Study through Caco-2 Cells Monolayer

Permeability evaluation was carried out using six- well plate Transwell inserts (polycarbonate membrane, 0.4 μm pore size, 24 mm diameter, Corning^®^, New York, NY, USA). Caco-2 cells were washed twice with Hank’s buffered salt solution (HBSS) for 15 min at 37 °C.

For the transport studies, Caco-2 cells were seeded on the Transwell inserts at a cell density of 1.5 × 10^5^ cells/cm^2^. Fresh growth medium (1.5 and 2.5 mL) was added in the apical and basolateral side, respectively and replaced every 2 days. Cells were allowed to grow and differentiate to confluent monolayers for 21 days post seeding. After 21 days in culture, cell monolayers were used for the following assays.

##### Ciprofloxacin Permeability through Caco-2 Cells Pretreatment with Ginsenoside Rg3

All cell monolayers were washed twice with Hank’s buffer. 0.02, 0.08 and 0.23 mg/mL of ginsenoside Rg3t were added to the apical compartment (1.5 mL) and Hank’s buffer was added to basolateral compartment (2.5 mL). After incubation for 120 min at 37 °C and 5% CO_2_ the cell monolayers were washed twice with Hank’s buffer and further and Hank’s buffer was added to apical and basolateral compartments and incubated and after 30 min of equilibration at 37 °C, transepithelial electrical resistance (TEER) of the monolayers was measured. Monolayers with values less than 220–250 Ω cm^2^ were discarded. The experiments were performed in the apical to basolateral (AP-BL) and basolateral to apical (BL-AP) directions. The AP-BL permeability experiment was started by adding ciprofloxacin-loaded optimized polymeric micelle, ciprofloxacin loaded-optimized formulation without G-Rg3, 0.02, 0.08 and 0.23 mg/mL of G-Rg3 in aqueous solution of labrafil + labrasol as controls on the apical site and Hank’s buffer on the basolateral site. 0.5 mL Sampling was done every 30 min up to 120 min after application from basolateral and replaced with Hank’s buffer. Samples were assayed by HPLC to determine ciprofloxacin concentration. The BL-AP permeability experiment was started by adding ciprofloxacin-loaded optimized polymeric micelle with and without G-Rg3 and controls on the basolateral site and Hank’s buffer on the apical site. Samples were taken from apical site every 30 min up to 120 min.

#### 3.2.11. Data Analysis

The apparent permeability coefficient (Papp) through Caco-2 cell monolayers was calculated with Equation. Q4% Percent of ciprofloxacin permeated through Caco-2 cell monolayers after 4 h (Q4%) was another parameter that calculated in this study:

P_app_ = (dc/dt)(V/C_0_A) (4)

In these equations dc/dt and dm/dt demonstrate change in concentration/mass of ciprofloxacin in the apical/basolateral sites, V is the volume of acceptor site, A is the surface area of cell monolayer (1.1 cm^2^) and C_0_ initial concentration of ciprofloxacin in donor compartment. Differences in permeability parameters was evaluated by Student’s unpaired *t*-test or one-way analysis of variance (ANOVA) (Minitab 16 software, Minitab Company, State College, PA, USA) at *p* <0.05.

## 4. Conclusions

As ciprofloxacin is a potent, widely used and safe antibiotic, and its effectiveness depends on its bioavailability, in this study the effect of polymeric micelles including ginsenoside on drug absorption and secretion through Caco-2 cells was evaluated. Prepared polymeric micelles demonstrated proper particle sizes below 190 nm with PDI for solubility in the gastrointestinal tract and permeability though the intestinal membrane. In addition, loading evaluations demonstrated that ciprofloxacin loading in polymeric micelle formulation is in the range of 27 to 88% and loading is strongly affected by the CMC concentration. An optimized formulation was prepared, characterized and then used for cellular studies. Based on the cellular results, the optimized polymeric micelles and G-Rg3 alone enhanced drug permeability through Caco-2 cells significantly in the absorptive direction. The G-Rg3 effect was dose dependent and the maximum effect was seen in 0.23 mg/mL concentration. Comparison of permeability parameters between the AP-BL with BL-AP directions showed that increasing in G-Rg3 concentration did not increase ciprofloxacin P and Q4% in the BL-AP direction. This means that P-gp may not be responsible for ciprofloxacin secretion into the gut. The main mechanism of ciprofloxacin transport through Caco-2 cells in both directions is active diffusion and. P-gp has inhibitory effect on ciprofloxacin permeability in the AP-BL direction that was blocked by ginsenoside. Although polymeric micelles without G-Rg3 increased drug secretion, G-Rg3 didn’t show this effect. This means that P-gp didn’t have inhibitory effects on ciprofloxacin secretion and the optimized polymeric micelles made from poloxamer after systemic administration can increase drug concentration in the gastrointestinal tract. Likewise, optimized polymeric micelles can increase ciprofloxacin intestinal solubility and absorption after oral administration.

## Figures and Tables

**Table 1:** Formulations components (concentrations are presented as gram unit) and their particle size and entrapment efficiency (EE%) (Mean ± SD, *n* = 5).

Batch No.	Chol	Leci	O.A	Polox	Lab + las (CMC)	PG	PEG	Cipro	R-g3	EE%	Particle Size (nm)
1	0.135	0.45	0.06	0.2	(3 CMC)	2	0.7	0.01	0.01	43.7 ± 5.7	137 ± 4.5
2	0.135	0.45	0.06	0.2	(3 CMC)	2	0.7	0.025	0.02	73.5 ± 6.6	121 ± 8.2
3	0.135	0.45	0.06	0.2	(3 CMC)	2	0.7	0.01	0.02	82.8 ± 5.8	120 ± 10.9
4	0.135	0.45	0.06	0.2	(3 CMC)	2	0.7	0.025	0.01	87.9 ± 7.3	127 ± 14.3
5	0.135	0.45	0.06	0.2	(1 CMC)	2	0.7	0.01	0.01	33.7 ± 3.7	183 ± 12.5
6	0.135	0.45	0.06	0.2	(1 CMC)	2	0.7	0.025	0.02	39.5 ± 4.2	126 ± 13.1
7	0.135	0.45	0.06	0.2	(1 CMC)	2	0.7	0.01	0.02	27.9 ± 3.1	109 ± 7.2
8	0.135	0.45	0.06	0.2	(1 CMC)	2	0.7	0.025	0.01	30.4 ± 2.4	153 ± 9.9

Chol: cholesterol; Leci: Lecithin; Polox: Poloxamer; Lab + Las: Labrafil + Labrasol; PG: propylene glycol; PEG: Polyethylene glycol; Cipro: Ciprofloxacin; R-g3: R-g3 Ginsenoside; EE%: entrapment efficiency.

**Table 2:** Optimized batch components, predicted and measured values of EE% and particle size.

	Surfactant + Co-Surfactant Concentration (mg/mL)	Ciprofloxacin Concentration (mg/mL)	Ginsenoside Concentration (mg/mL)
Batch Components	0.139	0.153	0.2
Batch characterization	EE%	Actual value	66.5
		Predicted value	63.3
		*p* value	0.461
	Particle Size (nm)	Actual value	135.3
		Predicted value	133.7
		*p* value	0.348
	D4%	Actual value	48.7
		Predicted value	46.2
		*p* value	0.394

**Table 3:** Transition temperature and enthalpy of optimized and blank formulations in heating program (mean ± SD, *n* = 3).

Formulation	Phase Transition Temperature (C^°^)	Phase Transition Enthalpy (mJ/mg)
Optimized formulation	−20 ± 3	742 ± 73
	0 ± 0.4	−100 ± 12
	140 ± 12	−723 ± 57
Optimized formulation without ginsenoside	−20 ± 3	189 ± 18
	0 ± 0.6	−185 ± 22
	140 ± 10	−160 ± 20
Blank formulation excluding ginsenoside	−20 ± 2	229 ± 25
	0 ± 0.3	−221 ± 13
	140 ± 9	−336 ± 44
Ciprofloxacin powder	260 ± 22	812 ± 69

**Table 4:** Permeability parameters of ciprofloxacin through Caco-2 cells monolayer in AP-BL and BL-AP directions (mean ± SD, *n* = 6).

Formulation	AP-BL Direction		BL-AP Direction	
	Q_4_%	P (cm/s) × 10^4^	Q_4_%	P (cm/s) × 10^4^
Optimized formulation	72 ± 1.9	2.40 ± 0.19	84.6 ± 5.1	2.89 ± 0.22
Optimized formulation without ginsenoside	58.3 ± 1.3	1.9 ± 0.16	79.2 ± 5.6	2.77 ± 0.28
Ciprofloxacin 0.14 mg/mL + ginsenoside 0.02 mg/mL (control 1)	47.2 ± 4.2	1.27 ± 0.11	65.5 ± 7.1	2.13 ± 0.15
Ciprofloxacin 0.14 mg/mL + ginsenoside 0.08 mg/mL (control 2)	60.7 ± 2.8	2.05 ± 0.14	67.7 ± 8.1	2.22 ± 0.32
Ciprofloxacin 0.14 mg/mL + ginsenoside 0.23 mg/mL (control 3)	77.3 ± 2.5	2.67 ± 014	64.7 ± 10.2	2.28 ± 0.3

## Supplementary Materials

The following are available online, Figure S1: Surface tension versus logarithm of lab + las concentration: (a) without polymer; (b) with polymer.
