# Supplementary material for: Permeability of Ciprofloxacin-Loaded Polymeric Micelles Including Ginsenoside as P-glycoprotein Inhibitor through a Caco-2 Cells Monolayer as an Intestinal Absorption Model
Source: Molecules. 2018 Jul 31;23(8):1904. doi: 10.3390/molecules23081904 (PMC6222528; doi:10.3390/molecules23081904)
Supplement: Supplementary file 1 [file molecules-23-01904-s001.pdf]

Supplementary Materials:

Permeability of Ciprofloxacin-Loaded Polymeric Micelles Including Ginsenoside as P-glycoprotein Inhibitor through a Caco-2 Cells Monolayer as an Intestinal Absorption Model

Behzad Sharif Makhmal Zadeh *, Golbarg Esfahani and Anayatollah Salimi

Nanotechnology Research Center, School of Pharmacy, Ahvaz Jundishapur University of Medical Sciences, Golestan Ave, Ahvaz 67123, Iran; anayatsalimi2003@yahoo.com; golbarg_isfahani@yahoo.com

* Correspondence: makhmalzadeh@yahoo.com; Tel.: +98-9163159706

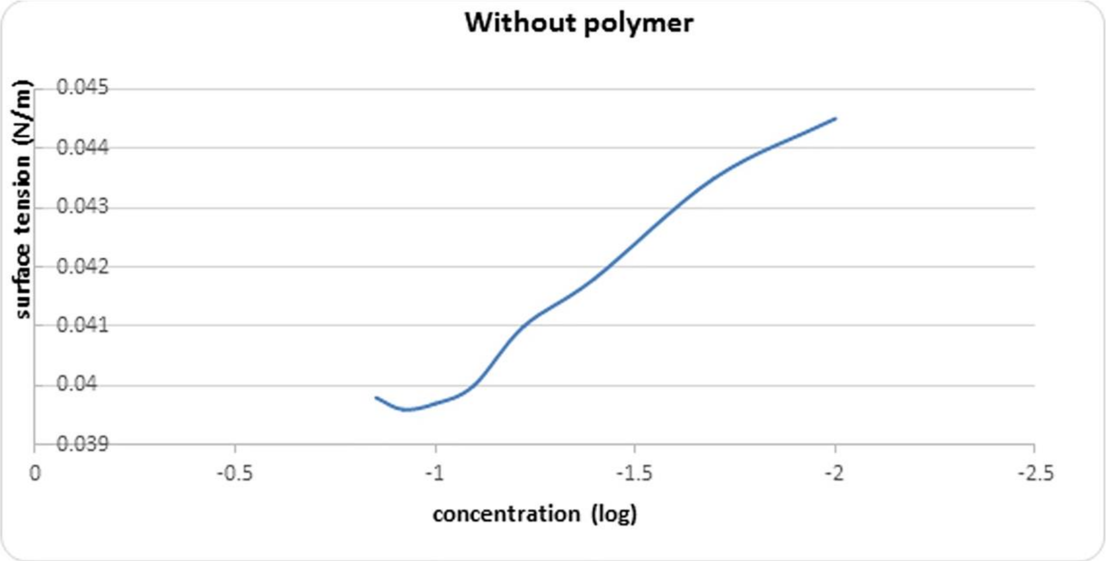

(**a**)

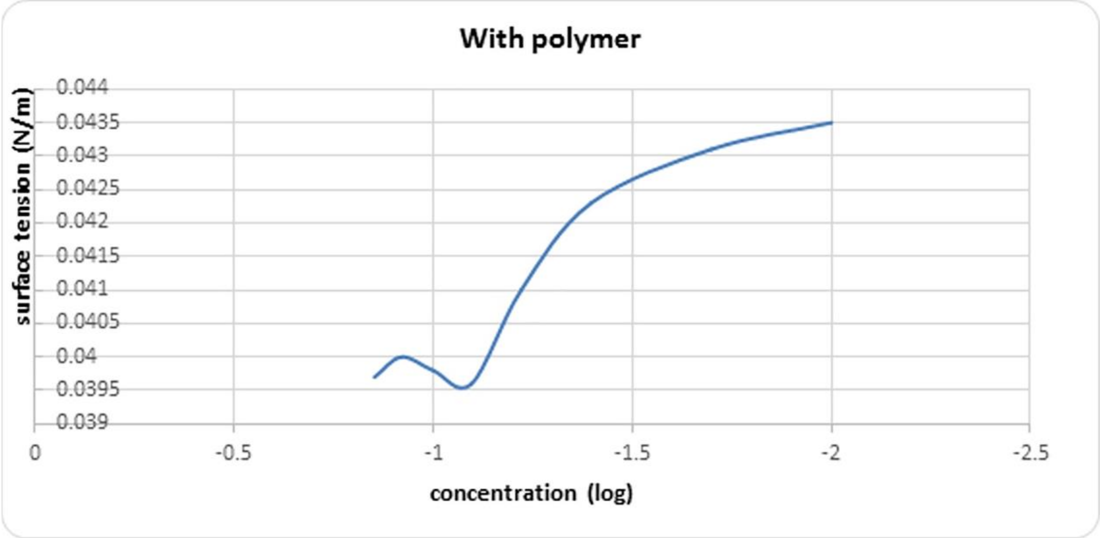

(**b**)

Figure S1. Surface tension versus logarithm of lab+las concentration: (**a**) without polymer; (**b**) with polymer.
